# Raman spectra simulation of antiplatelet drug-platelet interaction using DFT

**DOI:** 10.1038/s41598-024-51605-7

**Published:** 2024-01-16

**Authors:** Anna Kundalevich, Anastasia Kapitunova, Kirill Berezin, Andrey Zyubin, Ekaterina Moiseeva, Vladimir Rafalskiy, Ilia Samusev

**Affiliations:** 1 Immanuel Kant Baltic Federal University, REC “Fundamental and Applied Photonics”, Nanophotonics, Kaliningrad, 236016 Russia; 2 Institute of Physics, Saratov State University, Saratov, 410012 Russia; 3 Immanuel Kant Baltic Federal University, Kaliningrad, 236016 Russia

**Keywords:** Optical physics, Nanophotonics and plasmonics, Computer science

## Abstract

The paper reflects the results of molecular docking and mathematical DFT simulation for antiplatelet drugs and the target platelet receptor/ferment interaction in the limited area. The results of Raman spectra simulation are implemented and obtained from the interaction of the clopidogrel metabolite of the P2Y12 receptor. The interaction of aspirin with the COX-1 enzyme was also investigated. As a result, theoretical Raman spectra of the drug-receptor area were obtained. The theoretical data were compared with the experimental SERS results. The characteristic bands corresponding to metabolite/ferment and antiplatelet drug vibrations were clarified. The prospects of obtaining results for pathologies based on platelet conformations during cardiovascular diseases have been demonstrated.

## Introduction

Cardiovascular diseases (CVD) have remained the leading cause of death at the global level for decades worldwide. CVD total cases were doubled from 271 million in 1990 to 523 million in 2019, and the number of CVD deaths increased from 12.1 million in 1990, reaching 19.1 million in 2020^1^. The process of thrombus formation plays a crucial role in the detection, diagnosis, and treatment of CVD, and it is the object of close interest for scientists around the whole world^2^. The platelet and its structural changes investigations under the influence of internal and external factors still remain a challenging task today^3^. Raman spectroscopy and Surface-Enhanced Raman spectroscopy (SERS) can be very informative for platelets molecular structure investigation^4^. Raman-based spectroscopy methods used to perform the analysis of molecular components such as amino acids, proteins, lipids, etc.^5^ and can bring the understanding of the platelet structure and its spectral response to antiplatelet therapy, which is the key to personalized medicine. However, the obtained spectral data reflect information from a complex picture, including membrane elements, proteins, buffer solutions, substrates, etc. In this case, studying the key and target areas of drug influence on platelet elements makes sense. DFT-based methods are widely used for problems of computational chemistry and biochemistry. Both amino acids in bovine insulin and peptide of diamino acid l-aspartyl-l-glutamic acid in the zwitterionic state can be investigated using DFT^6,7^. Direct protein function^8^, riboflavin^9^, procainamide^10^ can be simulated and analyzed using DFT methods.

Due to the complexity of the spectral signatures obtained from the platelet mass, this paper presents the results of mathematical DFT modeling of limited antiplatelet drug and the target platelet receptor/ferment site. In particular, the results of modeling Raman spectra obtained from the results of the interaction of the clopidogrel metabolite on the P2Y12 receptor. The interaction of aspirin with the COX-1 enzyme was also investigated. As a result, theoretical Raman spectra of the drug-receptor area were obtained. The theoretical data were compared with the experimental SERS results.

## Methods

### Experimental methods

#### Platelets preparation

Twenty-three healthy volunteers were involved in the study. Written informed consent had been obtained from all healthy volunteers before any study procedures. All study documents, including informed consent and protocol were approved by Immanuel Kant Baltic Federal University Independence Local Ethic Committee (Protocol No 8, 16.05.2019). The methods used were carried out in accordance with the local ethics committee of the independence of the Immanuel Kant Baltic Federal University and in accordance with the Declaration of Helsinki. Healthy volunteers aged 18–45 years without acute and chronic diseases were included in the study. All healthy volunteers were divided into 3 groups: 11 subjects without antiplatelet therapy, 8 subjects after taking 100 mg aspirin, and 4 subjects after taking 300 mg clopidogrel. The preparation protocol was based on^11^. Briefly, fresh venous blood samples were taken from healthy volunteers in vacuum tubes containing EDTA (BD Vacutainer® spray-coated K2 EDTA Tubes). It was centrifuged at 60 g for 15 min to consequentially separate platelet-rich plasma (PRP) from red blood cells (RBC) and leukocytes. After that, the PRP was collected and placed in the new tube. Finally, platelets were collected by further centrifugation of the supernatant at 1500 g for 15 min. All the centrifugations were carried out at 4 °C using Eppendorf 5702R centrifuge. After platelet preparation, samples were immediately taken for examination by Surface-enhanced Raman spectroscopy (SERS).

#### SERS substrates fabrication

SERS substrate fabrication was performed in three steps: Firstly, in order to create roughness, anodization of 0.1 mm thick titanium films was carried out on the laboratory hand-made equipment with a current source and a galvanic bath, in which titanium electrodes were immersed. An aqueous solution of KOH (5%) was used as the electrolyte. Anodizing was carried out at a current density of j = 30 mA cm^−2^ for 5 min. After anodization, the titanium surface acquired a dark blue color. In the second stage, the gold nanoparticles were prepared by the femtosecond laser ablation method. In the third step, gold nanoparticles were deposited on these surfaces. The gold plate of 99.9% purity was placed in distilled water and AVESTA femtosecond laser unit with a TETA compressor (TETA Yb amplifier system) was used. The energy and pulse duration of the laser beam at λ = 1032 nm were 15 µJ and 280 fs, respectively. The repetition frequency and the number of pulses were set by an external generator. The solution volume in the cuvette was 1.2 ml, ablation time was 5 min; the thickness of the distilled water layer over the surface of the metal plate was 2 mm. Each package of laser pulses was focused to a new location of the plate. After ablation, the solution assumed a slightly red color. The hydrodynamic radius of the obtained nanoparticles was measured by dynamic scattering light method with PhotoCorr-Complex unit (LTD “PhotoCorr”, Russia) and was found to be in the range of 20–80 nm. As a last stage, deposition of ablative gold nanoparticles on titanium rough surfaces was carried out as follows: the titanium substrate was immersed in a gold nanoparticles solution, then the nanoparticles were deposited on the surface by evaporation of an aqueous colloidal gold solution at a temperature of 60 °C for 40 min. The detailed methodology of SERS substrates preparation was described in^12^.

#### SERS experiment

SERS spectra were obtained by Centaur U (LTD “NanoScanTechnology”, Russia) Raman spectrometer, using the λ = 532 nm DPSS Cobolt Samba excitation laser with 45 mW power on sample. The optical scheme included Olympus BX 41 microscope with 100X (NA 0.9) objective. Spectrometer Shamrock 750 (Andor, UK) had a focal length of 800 mm and was equipped with 300 gr mm^−1^ diffraction grating with 500 nm blaze. IDus 401-DV CCD camera (Andor, UK) with 1024 × 256 pixels sensor was used for the experiments. The spectrometer had a spectral resolution of 1.5 cm^−1^. The laser spot of 1 × 25 μm size was positioned at the platelets. Rayleigh scattering was eliminated by the notch filters. A 5 μl droplet of platelet-rich plasma was put on the substrate, dried for 5 min at room temperature, and then placed in the microscope holder. Three times averaged spectra from ten different places of the droplet have been collected for each sample. The signal acquisition time was 70 s. Each time before experiment, spectrometer was calibrated with silicon at a static spectrum centered at 520.1 cm^−1^ for 1 s. After registration, spectra were saved in .txt and specific format (.ngs) on the PC, connected to the Raman unit.

### Theoretical methods

#### Molecular docking

Molecular docking was performed using the Molecular Operating Environment (MOE) 2014 software platform. The binding site of the ligand and the receptor for further modeling were determined. The Gaussian software package was used for further calculations. Docking was performed on platelet P2Y12 and COX-1 receptors. Its structures were obtained from the Protein Data Bank (PDB). Based on a review of the literature (it was determined that the drugs that act on these receptors are clopidogrel (for P2Y12) and aspirin (for COX-1)). Literature sources indicate that P2Y12 is affected not by clopidogrel itself but by its active metabolite^13^, so it was used in molecular docking procedures. The structures of the ligands were obtained from the online libraries DrugBank and PubChem. Before implementing molecular docking procedures, platelet receptors were prepared. The amino acid sequence of the human platelet receptor P2Y12 (PDB: 4NTJ) was used as a query sequence to search for homologue models with known structures from the Protein Data Bank (PDB), and protein similarity was assessed using NCBI-BLAST^14^. For homologous modeling, a similar human P2Y12 protein (PDB: 4PZX) was chosen to complete the artifacts and gaps in the protein crystal structure. A three-dimensional model of the human receptor was created using the MODELER program (version 10.2). For COX-1 (PDB: 6Y3C) no homologous modeling has been performed. Next, the proteins were minimized first in the gas phase and then with a solvent, using the MMFF94x force field, to obtain the most stable conformation. After that, molecular docking of the protein and drug was performed.

#### Simulation of Raman Spectra

The Gaussian 16^15^ software package (license number: G64284555249899W-6922N) was used to calculate the theoretical Raman spectra. The Raman spectra were obtained by DFT using the selected functional (B3LYP)^16^ based on optimized molecular structures. The 6-31G(d) split valence bivalent base set was chosen as the basis set. Before theoretical calculations, the analyzed structures were energetically minimized in the MOE software package. Based on the results of molecular docking, the vibrational spectra of the interaction regions of platelet receptors P2Y12 and cyclooxygenase-1 (zwitterionic forms of amino acids) and metabolites of drugs inhibiting the receptors (thiol metabolite of clopidogrel H4 and aspirin, respectively) were mathematically calculated. In order to obtain more accurate theoretical results, linear scaling of the wave numbers was carried out. As a criterion for assessing the quality of calculation of oscillation frequencies, linear scaling of wave numbers^17^ was carried out according to the formula (1):

1 $$\frac{{v_{ex} }}{{v_{th} }} = av_{th} + b$$

where $v_{ex}$, $v_{th}$—are the experimental and calculated frequency (cm^−1^), *a* and *b* are coefficients to be determined. In the framework of this work, for the 6-31G(d) basis we used, the coefficients a = − 0.0000083526 and b = 0.98134 were chosen.

The Raman activities (Ai) calculated with the Gaussian 16 program were converted to relative Raman intensities (Ii) using the formula (2):

2 $$I_{i} = \frac{{f\left( {\nu_{0} - \nu_{i} } \right)^{4} A_{i} }}{{\nu_{i} \left[ {1 - e^{{ - \frac{{hc\nu_{i} }}{kT}}} } \right]}}$$

where $v_{0}$ is the exciting frequency (cm^−1^), $v_{i}$ is the vibrational frequency (cm^−1^) of the ith normal mode, $h$, $c$, and $k$ are fundamental constants, and $f$ is a suitably chosen common normalization factor for all peak intensities. The calculated spectra were reported by assigning to each normal mode a Lorentzian shape with a 5 cm^−1^ full width at half-maximum. For these calculations, a program was used for modeling and visualizing mixed IR and Raman spectra based on quantum mechanical calculation data^18^.

#### Comparison of theoretical and experimental spectral Raman data

Experimental data were divided into samples: healthy patients, healthy patients on aspirin therapy and healthy patients on clopidogrel therapy. Using the laboratory build Parcer analyzer, statistical processing was carried out. The data recorded from the device were converted into .txt and .csv formats. Further, the data were entered into tables of certain groups and a fixed frequency grid was created with a step of 5 cm^−1^. The Parcer analyzer decomposes the spectrum into grid cells in the range from 400 to 1800 cm^−1^ in 5 cm^−1^ steps. Thus, all spectral fluctuations were correlated with the designated grid. Vibrational bands were used to correlate the obtained experimental data with the calculated theoretical ones. In the Origin 2021 program, experimental platelet spectra of healthy patients and patients on therapy normalized to 100 were built, after which vibrational modes were added.

## Results

**Figure 1:**
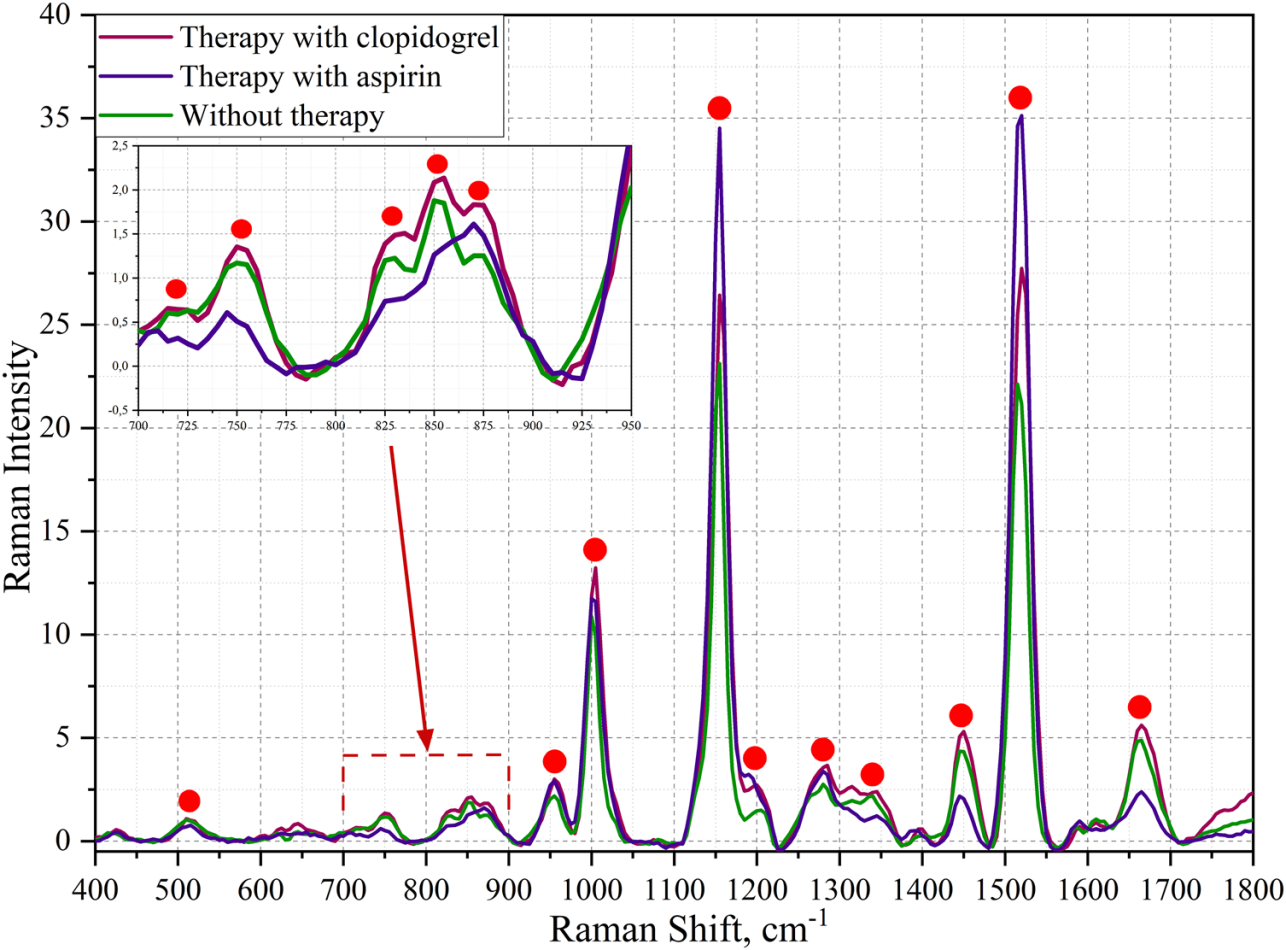
Comparison of SERS spectra of platelets from a healthy person without therapy, on aspirin therapy and on clopidogrel therapy. Red markers indicate main experimental characteristic bands.

Experimental SERS spectra were obtained from the platelets of healthy volunteers, a healthy volunteer during clopidogrel therapy, and healthy volunteers during aspirin therapy. For a correct comparison of the results obtained, an analysis of three groups of platelets from one patient was carried out. Vibrational bands were used to correlate the obtained experimental data with the calculated theoretical ones. Figure 1 shows the Raman spectra for three groups of healthy patients: on aspirin therapy, on clopidogrel therapy, and without therapy.

**Figure 2:**
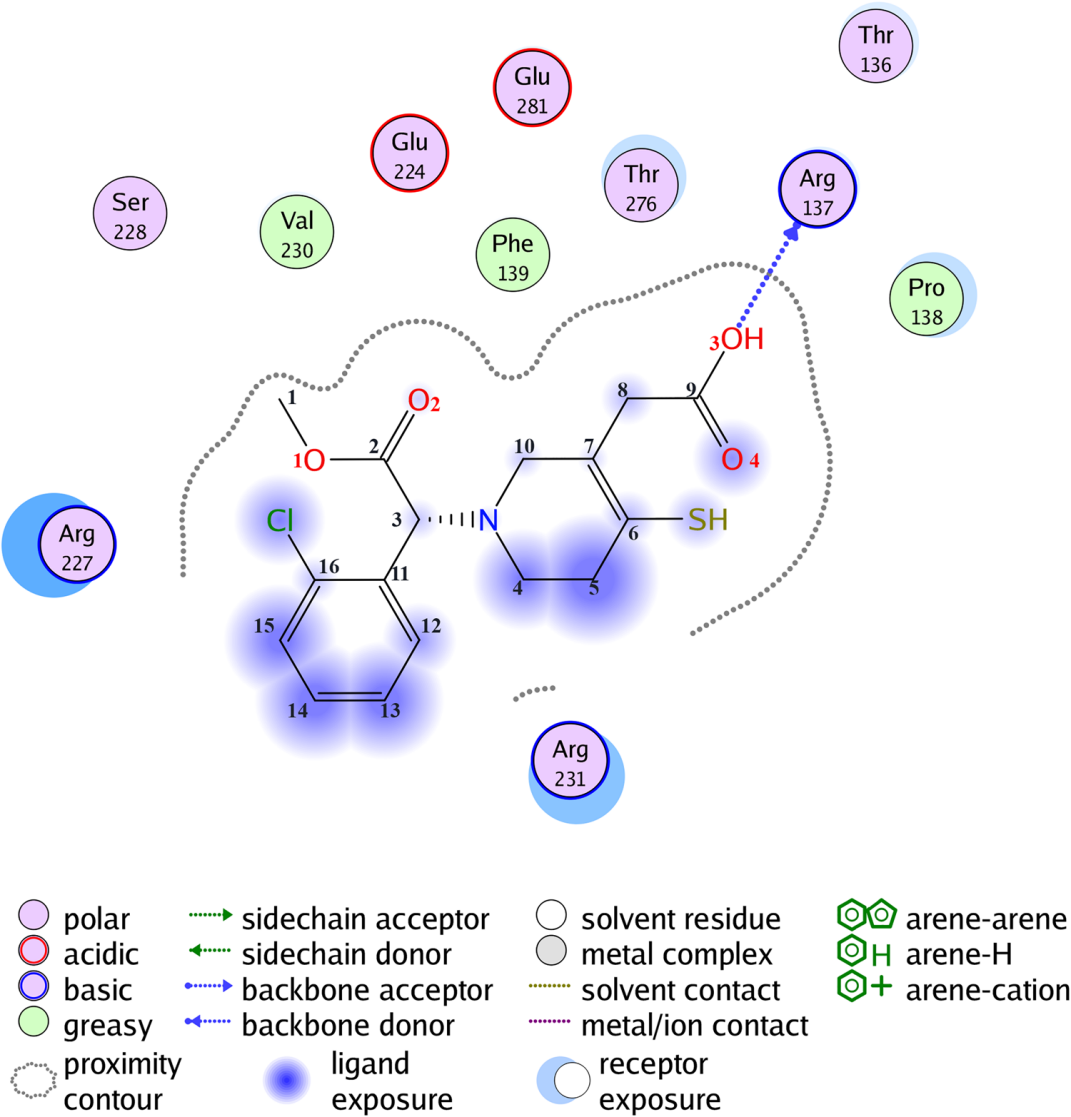
Interaction diagram of the P2Y12 receptor and clopidogrel metabolite.

Docking calculation of the P2Y12 receptor and the clopidogrel metabolite H4 was performed as the first step. As a result, diagrams of the ligand–protein interaction were obtained. The software performed 1000 iterations of ligand-to-protein attachment and sorted them by interaction enthalpy. However, variants of the docking of the metabolite and the receptor with a minimum energy were further considered (Fig. 2), since this interaction variant is the most probable.

**Figure 3:**
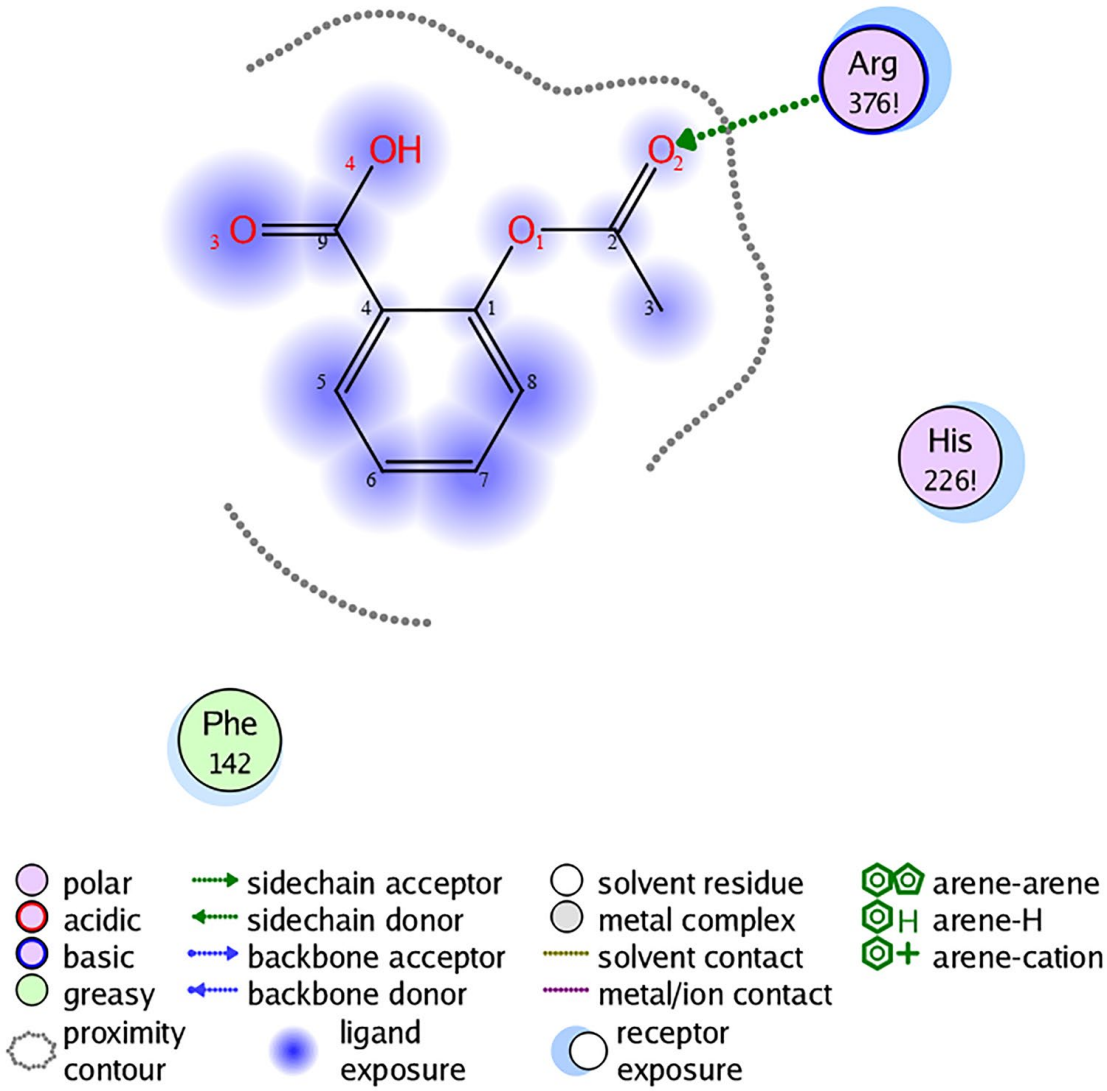
Interaction diagram of the COX-1 receptor and aspirin.

Analyzing the obtained data, the surface contact interactions of the protein and the ligand were determined, however, these types of interactions did not form bonds and therefore were not used in further analysis. Also, the data obtained indicate that the binding of the metabolite occurs with Arginine at position 137 due to the donor–acceptor interaction, where the donor is the oxygen of the metabolite, the acceptor is the amino acid (Fig. 2). The cyclooxygenase-1 receptor and aspirin system were used for molecular docking implementation as a next step. In the course of molecular docking, diagrams of the ligand–protein interaction were obtained. The software performed 1000 iterations of ligand-to-protein attachment and sorted them by interaction enthalpy. However, variants of the docking of the metabolite and the receptor with a minimum energy were further considered (Fig. 3), since this variant of interaction is the most probable.

It was shown that aspirin binds to arginine at position 376 through a donor–acceptor bond, where the metabolite is the donor and the amino acid is the acceptor (Fig. 3).

In the third step, Raman spectra for the selected chemical compounds in the range of 0–4000 cm^−1^ were calculated using the Gaussian program. After that, linear scaling of the wave numbers was carried out in the area of the fingerprint 400–1800 cm^−1^.

**Figure 4:**
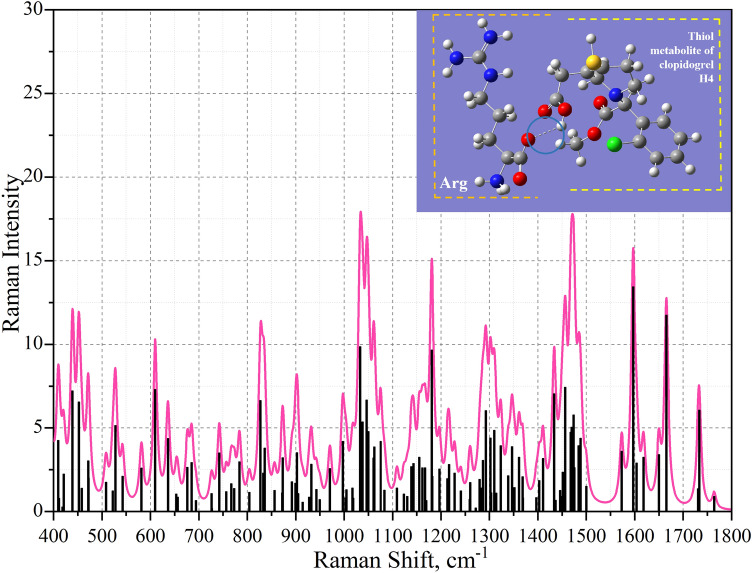
Theoretical Raman spectrum of the active metabolite of clopidogrel and arginine after frequency adjustment.

**Figure 5:**
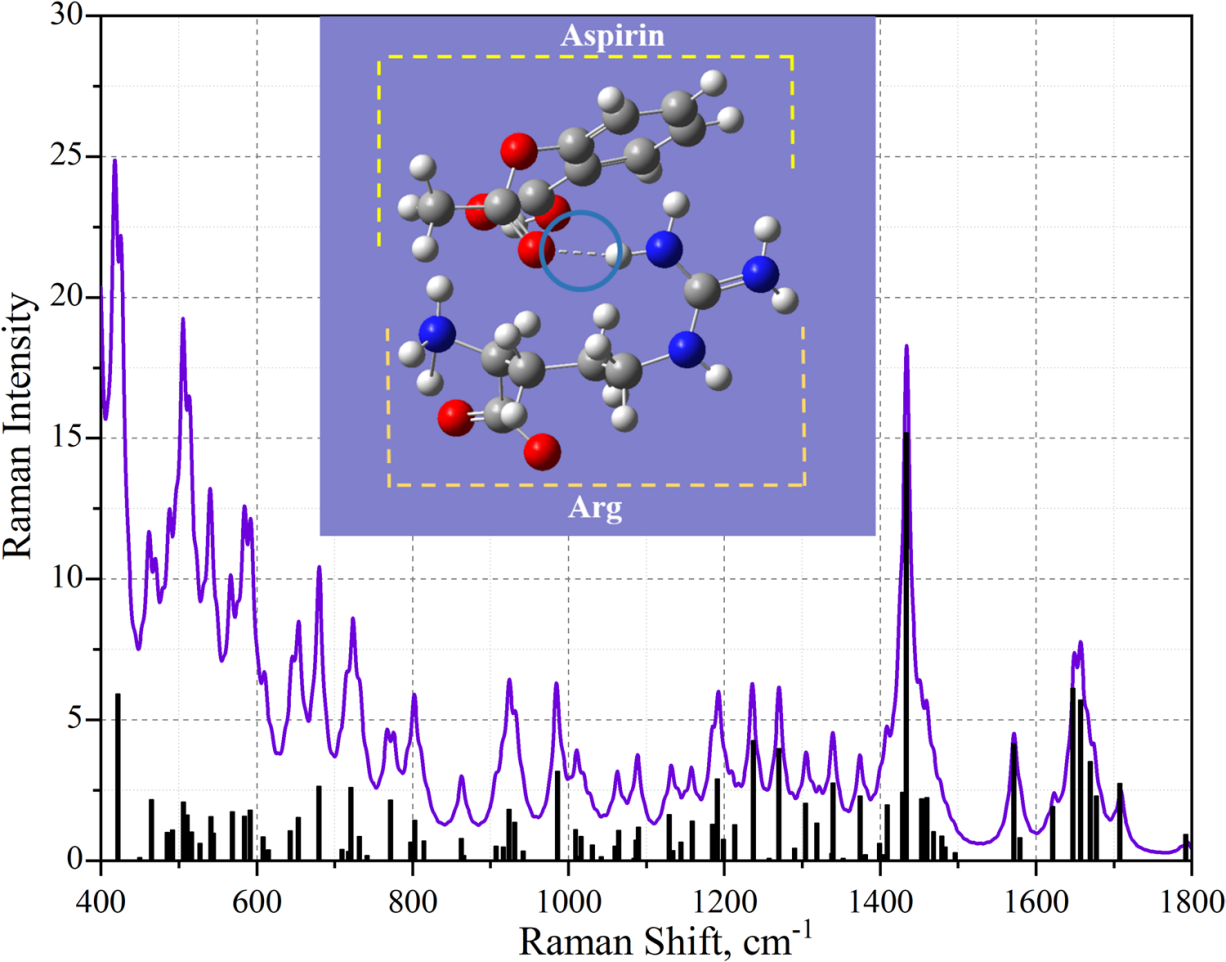
Theoretical Raman spectrum of aspirin and arginine after frequency adjustment. Results of correlating theoretical data with experimental data.

The results of the calculated Raman spectra are presented in Fig. 4 (the active metabolite of clopidogrel and arginine) and Fig. 5 (aspirin and arginine).

**Figure 6:**
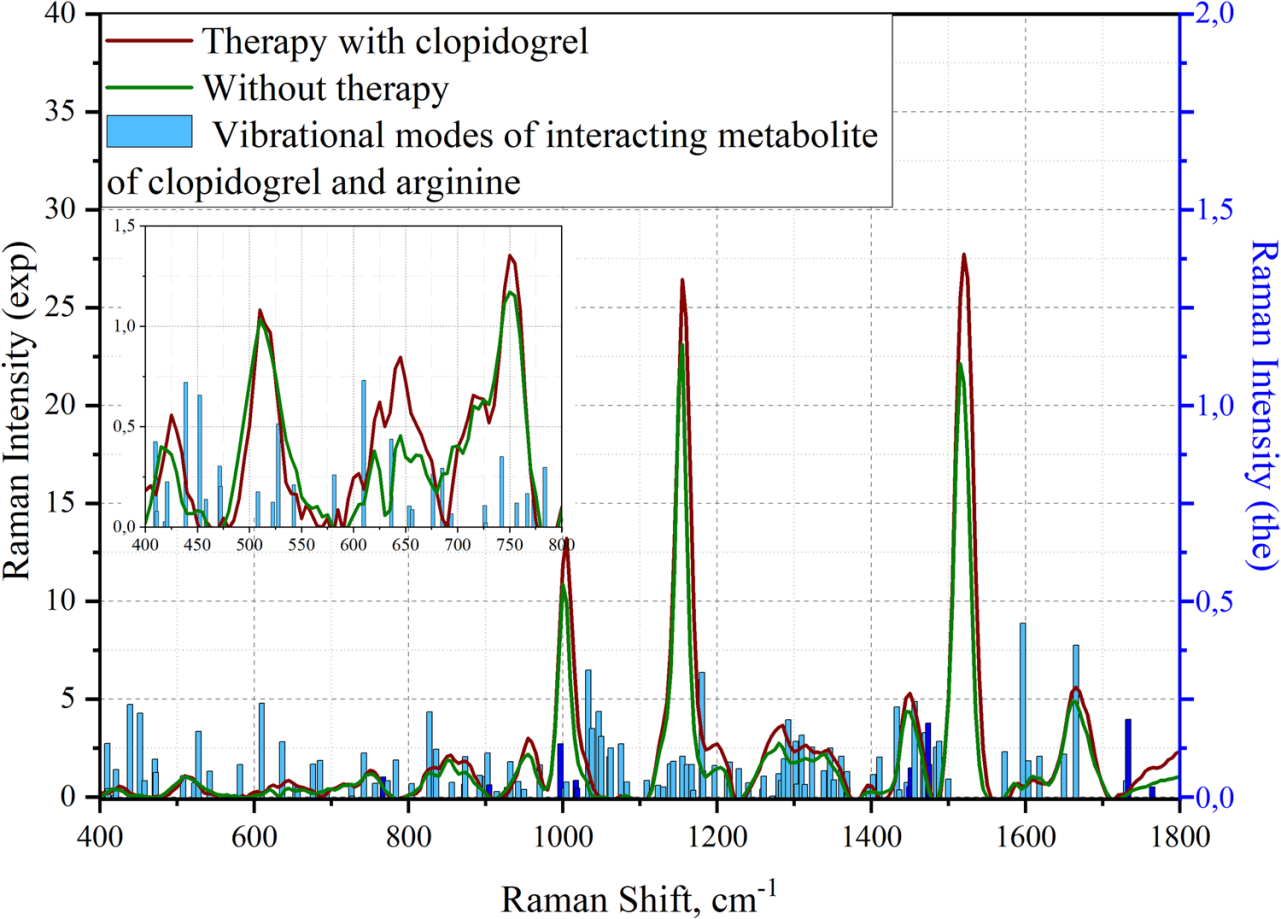
Superimposed modes of the metabolite of clopidogrel and arginine on the experimentally obtained spectra of platelets from different samples. The left scale shows the intensity of vibrational modes of the experimental data, the scale on the right shows the theoretically calculated vibrational modes. The vibrational modes presented in Table 3 are highlighted in blue.

The comparison of the experimental and theoretical spectra was performed as a last step. In the case of considering the theoretically obtained spectra of the binding site of the P2Y12 receptor and the clopidogrel thiol metabolite H4, a comparison was made between healthy patients without therapy and with clopidogrel therapy (Fig. 6).

Using the Gaussian 16 software package, the vibrational modes of the theoretical spectrum of the metabolite of clopidogrel and arginine were deciphered. Since the obtained calculated data correlates with the experimental data, the area of the fingerprint was considered. The results were entered in Table 1.

**Table 1:** Calculated wavenumbers and vibrational mode assignments for the interaction site of clopidogrel thiol metabolite H4 and arginine at the P2Y12 receptor.

Vibrational mode number	Wave number, cm^−1^	Raman strength	Molecule	Oscillation type
46	397	0.05	Arginine	Rocking H-N1-H
47	410	0.04	Metabolite	Rocking H-C10-H
48	411	0.49	Arginine	Rocking H-N1-H and scissoring N3-C5-N4
49	419	0.13	Metabolite	Rocking H-C8-H and O3-C9 = O4
50	421	0,08	Metabolite	Ring deformation of 1-azacyclohexnene-3
51	439	0.10	Arginine	Wagging N3-C5-N4 and rocking H-N4-H
52	452	0.03	Metabolite	Deformation of the aromatic ring out of its plane and deformation of the 1-azacyclohexnene-3 ring
53	458	0.10	Metabolite	Deformation of the aromatic ring out of its plane and deformation of the 1-azacyclohexnene-3 ring
54	471	0,02	Metabolite	Deformation of the aromatic ring out of its plane and deformation of the 1-azacyclohexnene-3 ring
55	472	0.42	Arginine	Scissoring C2-C3-C4 and N2-C5-N4
56	508	0.27	Metabolite	Wagging C7=C6-C5 in the 1-azacyclohexnene-3 and scissoring C6-S-H
57	522	0.12	Metabolite	Out-of-plane deformation of the aromatic ring
58	528	0.12	Arginine	Scissoring N2-C5-N3
59	543	0.08	Arginine	Scissoring C2-C1-N1
60	581	0.03	Metabolite	Stretching of aromatic and 1-azacyclohexnene-3 rings and scissoring O3-C9=O4
61	610	0.09	Metabolite	Stretching of aromatic and 1-azacyclohexnene-3 rings and scissoring O3-C9=O4
62	636	0.20	Metabolite	Stretching of aromatic and 1-azacyclohexnene-3 rings and scissoring O3-C9=O4
63	653	0.12	Metabolite	Aromatic ring stretching and rocking H-C4-H, H-C5-H
64	656	0.11	Arginine	Rocking H-C2-H, scissoring C2-C1-N1 and O2-C6=O1
65	676	0.04	Metabolite	Aromatic ring stretching
66	685	0.09	Arginine	Scissoring H-N2-C5, rocking H-C4-H and wagging C4-N2-H
67	694	0.22	Metabolite	Deformation of the aromatic ring out of its plane, rocking H-C4-H, H-C5-H
68	726	0.02	Metabolite	Aromatic ring stretching, rocking C11-C2-C3
69	727	0,12	Arginine	Rocking H-C2-H, H-C3-H and wagging O2-C6=O1
70	742	0.09	Metabolite	Vibration of the hydrogen atoms of an aromatic ring out of its plane
71	757	0.09	Arginine	Rocking H-C2-H, H-C3-H, H-C2-H, H-C4-H
**72**	**767**	**0.07**	**–**	**Rocking H-C2-H, H-C3-H, H-C2-H, H-C4-H (arginine) and scissoring C7-C8-C9, vibration of hydrogen atoms out of the plane of the aromatic ring (metabolite)**
73	773	0.12	Metabolite	Scissoring C7-C8-C9, vibration of hydrogen atoms out of the plane of the aromatic ring, rocking H-C4-H, H-C5-H
74	784	0.06	Arginine	Wagging H-N3-H, H-N4-H
75	804	0.21	Arginine	Rocking H-C2-H, H-C3-H, H-C2-H, H-C4-H and scissoring O2-C6=O1
76	827	0.13	Metabolite	Rocking H-C4-H, H-C5-H in 1-azacyclohexnene-3 and scissoring C2-C3-O1
77	833	0.07	Metabolite	Stretching of 1-azacyclohexnene-3 and scissoring C-S–H
78	836	0.07	Arginine	Wagging H-N3-H
79	856	0.11	Metabolite	Vibration of hydrogen atoms out of the plane of the aromatic ring
80	872	0.06	Arginine	Rocking H-C2-H, H-C3-H, H-C4-H and stretching C5-N2, C5-N3, C5-N4
81	873	0.04	Metabolite	Stretching of 1-azacyclohexnene-3, vibration of hydrogen atoms out of the plane of the ring, stretching C4-C5-C6
82	892	0.17	Metabolite	Stretching O3-C9-C8
83	900	0.05	Metabolite	Wagging H-O3-C9
84	902	0.13	Metabolite	Scissoring H–S-C6 and stretching of 1-azacyclohexnene-3
**85**	**905**	**0.15**	**–**	**Twisting H-C2-H, H-C3-H, H-C4-H, stretching C5-N3, C5-N4 (arginine) and wagging H-O3-C9 (metabolite)**
86	915	0.03	Metabolite	Rocking H-C4-H, H-C5-H, H-C8-H, H-C10-H
87	928	0.02	Metabolite	Vibration of hydrogen atoms in the aromatic ring, scissoring H-S-C6
88	932	0.14	Metabolite	Scissoring H-S-C6, rocking H-C4-H, H-C5-H, H-C8-H, H-C10-H
89	943	0.09	Arginine	Twisting H-C2-H, H-C3-H, H-C4-H, wagging H-N1-H and H-O2-C6
90	950	0.00	Arginine	Wagging H-N1-H and H-O2-C6
91	963	0.10	Metabolite	Vibration of hydrogen atoms in the aromatic ring
92	971	0.15	Metabolite	Stretching of 1-azacyclohexnene-3 and rocking H-C4-H, H-C5-H, H-C8-H, H-C10-H
**93**	**997**	**0.07**	**–**	**stretching of 1-azacyclohexnene-3 and scissoring H-S-C6 (metabolite) and wagging N1-H(arginine)**
94	1003	0.03	Arginine	Wagging N1-H and H-O2-C6
95	1004	0,09	Metabolite	Stretching of 1-azacyclohexnene-3, rocking H-C4-H, H-C5-H, H-C10-H
**96**	**1017**	**0.09**	**–**	**Stretching C2-C3-C4 (arginine) and stretching C1-O1-C2 (metabolite)**
**97**	**1019**	**0.52**	**–**	**Stretching C2-C3-C4 (arginine) and stretching C1-O1-C2 (metabolite)**
98	1033	0.22	Metabolite	Aromatic ring stretching
99	1038	0.17	Arginine	Stretching C2-C3-C4
100	1046	0.14	Arginine	Stretching C2-C3-C4 and rocking H-N1-H, H-N3-H, H-N4-H
101	1050	0.07	Metabolite	Aromatic ring stretching and rocking H-C4-H
102	1061	0.11	Metabolite	Rocking H-C4-H, H-C5-H in 1-azacyclohexnene-3
103	1062	0.09	Arginine	Stretching C2-C3-C4 and rocking H-C2-H, H-C3-H, H-C4-H
104	1075	0.06	Metabolite	Stretching of 1-azacyclohexnene-3, scissoring C6-S-H, stretching N-C4-C5
105	1083	0.13	Arginine	Rocking H-N3-H, H-N4-H
106	1109	0.02	Arginine	Twisting H-C2-H, H-C3-H, H-C4-H and stretching C3-C4-N2
107	1123	0.07	Metabolite	Vibration of hydrogen atoms in the plane of the aromatic ring, twisting H-C4-H, H-C5-H
108	1130	0.27	Arginine	Rocking H-N3-H, H-N4-H and stretching C5-N2-C4
109	1139	0.06	Metabolite	Vibration of hydrogen atoms in the plane of the ring, twisting H-C5-H, stretching C3-N-C10
110	1143	0.10	Metabolite	Rocking H-C1-H
111	1155	0.01	Metabolite	Rocking H-C9-H, twisting H-C10-H
112	1161	0.17	Metabolite	Vibration of hydrogen atoms in the plane of the aromatic ring, twisting H-C8-H, stretching C-N-C
113	1167	0.07	Metabolite	Vibration of hydrogen atoms in the plane of the aromatic ring
114	1169	0.08	Arginine	Twisting H-C2-H, H-C3-H, H-C4-H and H-N3-H, H-N4-H
115	1181	0.13	Metabolite	Stretching of the aromatic ring, vibration of hydrogen atoms in the plane of the ring, stretching N-C3-C11
116	1182	0.27	Metabolite	Stretching C8-C7-C10, twisting H-C5-H
117	1197	0.09	Metabolite	Twisting H-C5-H, H-C4-H
118	1213	0.05	Metabolite	Wagging H-C1-H, H-C2-H, H-C3-H, H-C4-H, rocking H-N1-H
119	1217	0.03	Arginine	Wagging H-C1-H, H-C2-H, H-C3-H, H-C4-H
120	1228	0.06	Arginine	Rocking H-C4-H, H-C5-H, H-C10-H, stretching C2-C3-C11
121	1241	0.03	Metabolite	Rocking H-C4-H, H-C10-H, stretching C2-C3-C11
122	1259	0.11	Metabolite	Scissoring H-O1-C2 and C2-C3-H
123	1260	0.07	Metabolite	Rocking H-C2-H, H-C3-H, H-C4-H and H-N1-H
124	1272	0.06	Arginine	Rocking H-C4-H, H-C5-H, H-C10-H
125	1280	0.13	Metabolite	Wagging H-C2-H, H-C3-H, H-C4-H and twisting H-N1-H
126	1283	0.16	Arginine	Twisting H-C4-H, H-C5-H, H-C10-H and aromatic ring deformation
127	1287	0.11	Metabolite	Twisting H-C3-H, H-C4-H, H-C5-H, H-C10-H and aromatic ring deformation
128	1293	0.32	Metabolite	Rocking H-C2-H, H-C3-H, H-C4-H and stretching N2-C5, N3-C5, N4-C5
129	1302	0.07	Arginine	Wagging H-C8-H
130	1303	1.23	Metabolite	Twisting H-C2-H, H-C3-H, H-C4-H
131	1310	0.37	Arginine	Scissoring C2-C3-H and twisting H-C3-H
132	1314	0.08	Metabolite	Wagging H-C2-H, H-C3-H, H-C4-H, scissoring H-C2-N
133	1323	0.05	Arginine	Stretching N2-C5, N3-C5, N4-C5, twisting H-N3-H, wagging H-C3-H
134	1339	0.05	Arginine	Wagging H-C5-H, twisting H-C4-H
135	1346	0.05	Metabolite	Stretching C8-C7-C10, twisting H-C5-H
136	1352	0.29	Arginine	Rocking H-N1-H, scissoring H-C2-C1, twisting H-C2-H
137	1361	0.07	Metabolite	Wagging H-C4-H and N-C3-H
138	1369	0.03	Metabolite	Wagging H-C3-H, H-C10-H, scissoring C11-C3-H
139	1369	0.03	Arginine	Wagging H-C2-H, H-C3-H, H-C4-H, scissoring H-C5-N3
140	1397	0.05	Arginine	Wagging H-C2-H, H-C3-H, H-C4-H
141	1403	0.18	Metabolite	Scissoring H-O1-C1 and H-C8-H
142	1411	0.01	Metabolite	Wagging H-C10-H, H-C4-H
143	1433	0.36	Arginine	Scissoring H-N2-C4
144	1437	0.07	Metabolite	Vibration of hydrogen atoms in the plane of the ring
145	1446	0.18	Metabolite	Scissoring H-C1-H
**146**	**1450**	**0.13**	**–**	**scissoring H-O2-C6 (arginine) and scissoring H-C9-H (metabolite)**
**147**	**1451**	**0.20**	**–**	**scissoring H-O2-C6 (arginine) and scissoring H-C9-H (metabolite)**
148	1456	0.16	Metabolite	Scissoring H-C5-H
149	1468	0.16	Metabolite	Scissoring H-C10-H
150	1470	0.11	Metabolite	Scissoring H-C1-H
**151**	**1474**	**0.03**	**–**	**Scissoring H-C2-H, H-C3-H (arginine) and scissoring H-C1-H (metabolite)**
**152**	**1475**	**0.00**	**–**	**Vibration of hydrogen atoms in the plane of the aromatic ring (metabolite) and scissoring H-C2-H, H-C3-H (arginine)**
**153**	**1475**	**0.12**	**–**	**Scissoring H-C1-H (metabolite) and scissoring H-C2-H, H-C3-H (arginine)**
154	1484	0.13	Arginine	Scissoring H-C2-H, H-C3-H, H-C4-H
155	1488	0.31	Metabolite	Scissoring H-C10-H, H-C4-H
156	1500	0.07	Arginine	Scissoring H-C2-H, H-C3-H, H-C4-H
157	1573	0.06	Metabolite	Deformation of the aromatic ring in the ring plane
158	1597	0.38	Metabolite	Deformation of the aromatic ring in the ring plane
159	1604	0.05	Arginine	Scissoring H-N3-H, H-N4-H
160	1618	0.03	Arginine	Scissoring H-N3-H, H-N4-H
161	1650	0.06	Arginine	Scissoring H-N1-H
162	1665	1.12	Metabolite	Seformation of 1-azacyclohexnene-3
**163**	**1731**	**0.53**	**-**	**Stretching C2=O2 (metabolite)** **and scissoring H-N1-H (arginine)**
**164**	**1733**	**0.84**	**-**	**Stretching C2=O2, scissoring C9-O3-H (metabolite) and stretching C6=O1 (arginine)**
**165**	**1764**	**0.37**	**-**	**Stretching C2= O2 (metabolite) and stretching C6=O1, scissoring C9-O3-H (arginine)**

Significant values are in [bold].

Calculated wavenumbers are presented with integer precision. The atomic number is indicated after the name of the element.

By superimposing theoretically calculated vibrational modes on the experimentally obtained spectra of platelets from a person who did not undergo therapy, and on the spectra of a person who is under clopidogrel therapy, it can be determined that the changes occurring in the P1Y12 receptor can be explained by the binding of the clopidogrel metabolite to arginine at position 137 in the receptor. It can be seen from the graphs that the change in the intensities and spectral shifts of the spectra during therapy correlate with the theoretically calculated vibrational modes.

**Figure 7:**
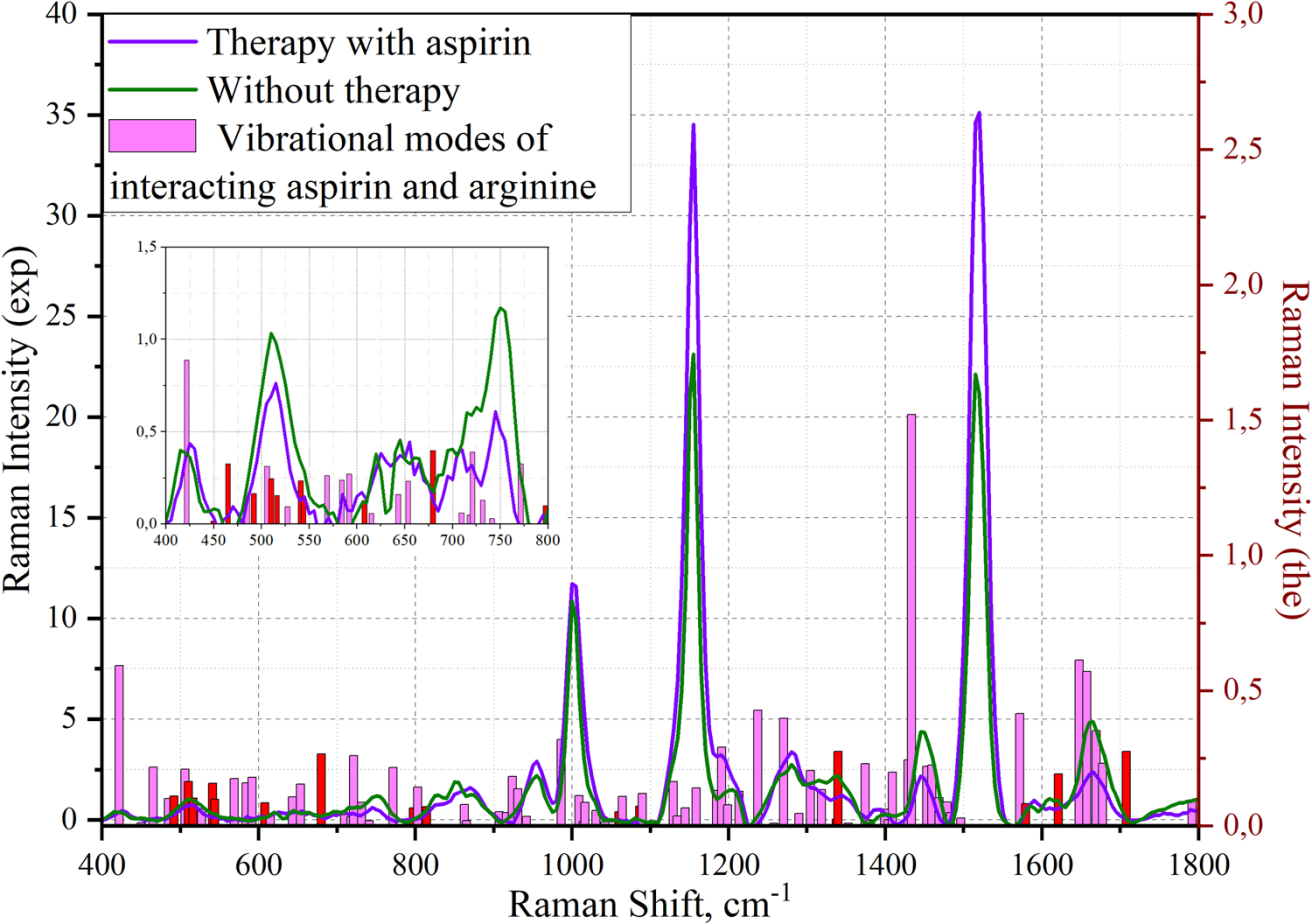
Superimposed modes of aspirin and arginine on the experimentally obtained spectra of platelets from different samples. The scale on the left shows the intensity of vibrational modes of the experimental data, the scale on the right shows the theoretically calculated vibrational modes. The vibrational modes presented in Table 3 are highlighted in red.

In the case of considering the theoretically obtained spectra of the binding site of the COX-1 receptor and aspirin, a comparison was made of healthy patients without therapy and with aspirin therapy (Fig. 7).

Using the Gaussian software package, the vibrational modes of the theoretical spectrum of aspirin and arginine were deciphered. Since the obtained calculated data correlates with the experimental data, the area of the fingerprint was considered. The results are reflected in Table 2.

**Table 2:** Calculated wave numbers and assignment of vibrational modes for the site of interaction between aspirin and arginine in the COX-1 receptor.

Vibrational mode number	Wave number, cm^−1^	Raman strength	Molecule	Oscillation type
30	391	0.54	Aspirin	Deformation of the aromatic ring out of the plane of the ring
31	422	0.59	Aspirin	Scissoring O3=C9-C4 and aromatic ring stretching
32	450	0.01	–	Twisting H-N1-H, scissoring C1-C2-C3 (arginine) and scissoring C9-O4-H (aspirin)
33	465	0.22	–	Rocking H-N1-H (arginine) and rocking H-O4-C9 (aspirin)
34	485	0.10	Arginine	Wagging H-N4-H
35	492	0.11	–	Rocking H-N1-H (arginine) and rocking H-O4-C9 (aspirin)
36	506	0.21	Aspirin	Twisting H-O4-C9 and deformation of the aromatic ring out of the plane of the ring
37	510	0.16	Arginine	Wagging H-N4-H
38	516	0.10	–	Wagging H-N4-H (arginine) and deformation of the aromatic ring in the ring plane (aspirin)
39	527	0.06	Arginine	Scissoring N4=C5-N2 and rocking H-C3-H
40	541	0.16	–	Deformation of the aromatic ring in the ring plane, scissoring O4-C9=O3 (aspirin) and rocking H-C3-H (arginine)
**41**	**544**	**0.10**	**–**	**Scissoring O3=C9-C4 and aromatic ring stretching**
42	569	0.17	Aspirin	Wagging O1=C2-O2
43	584	0.16	–	Scissoring N-C-N and N-C-C (arginine) and aromatic ring deformation and scissoring N2-C5-N3 (arginine)
44	592	0.18	Aspirin	Out-of-plane deformation of the aromatic ring
45	608	0.08	Aspirin	Deformation of the aromatic ring in the ring plane, scissoring O4-C9=O3
46	615	0.04	Arginine	Scissoring N3-C5-N2
47	643	0.11	Aspirin	Vibration of hydrogen atoms out of the plane of the ring
48	653	0.15	Aspirin	Aromatic ring stretching, scissoring O1-C2=O2 and wagging O4-C9=O3
49	680	0.27	–	Aromatic ring stretching (aspirin) и rocking H-N3-H (arginine)
50	709	0.04	Arginine	Wagging N3-C5=N4 and twisting H-N3-H
51	718	0.03	Arginine	Rocking H-C2-H and O2=C6-O1
52	721	0.26	Aspirin	Aromatic ring stretching and stretching O4-C9-C4
53	731	0.09	Arginine	Twisting H-N3-H
54	741	0.02	Arginine	Rocking H-C2-H, H-C3-H, H-C4-H
55	771	0.22	Aspirin	Vibration of hydrogen atoms out of the plane of the ring
**56**	**797**	**0.07**	**–**	**Wagging H-N3-H (arginine) and change in hydrogen bond length**
57	803	0.14	Arginine	Rocking H-C2-H, H-C3-H, H-C4-H and wagging H-N3-H
58	814	0.07	–	Aromatic ring stretching (aspirin) and wagging H-N3-H (arginine)
59	862	0.08	Aspirin	Wagging H-C8-C7
60	865	0.02	Arginine	Twisting H-C2-H, H-C3-H, H-C4-H
61	907	0.05	Arginine	Scissoring H-N1-H
62	916	0.05	Aspirin	Vibration of hydrogen atoms out of the plane of the ring
63	924	0.18	Aspirin	Scissoring O1-C2, C2=O2 и C2-C3
64	931	0.14	Arginine	Twisting H-C4-H and stretching N2-C5-N3 and N2-C5=N4
65	942	0.03	Arginine	Wagging H-N1-H and stretching N1-C1
66	986	0.32	Aspirin	Aromatic ring stretching
67	1009	0.11	Aspirin	Deformation of the aromatic ring in the ring plane and stretching C9-O4
68	1014	0.02	Aspirin	Wagging H-C3-H
69	1016	0.09	Arginine	Stretching C2-C3-C4 and wagging H-N1-H
70	1031	0.06	Arginine	Stretching C2-C3-C4
71	1042	0.01	Aspirin	Wagging H-C3-H
**72**	**1060**	**0.05**	**–**	**Rocking H-N3-H and change in hydrogen bond length**
73	1064	0.11	Arginine	Rocking H-N3-H
74	1084	0.01	Arginine	Stretching C1-C2 and rocking H-N3-H
75	1087	0.07	–	Rocking H-N4-H, H-N3-H (arginine) and vibration of hydrogen atoms in the plane of the ring
76	1090	0.12	Arginine	Rocking H-N4-H, H-N3-H
77	1129	0.16	Aspirin	Vibration of hydrogen atoms in the plane of the ring
78	1134	0.04	Arginine	Stretching N1-C1-C2 and rocking H-C3-H, H-C2-H
79	1144	0.07	Arginine	Rocking H-N3-H, H-N4-H and stretching C4-N2-C5
80	1159	0.14	Aspirin	Vibration of hydrogen atoms in the plane of the ring
81	1185	0.13	Arginine	Twisting H-C4-H and H-N1-H
82	1191	0.29	Aspirin	Scissoring H-O4-C9
83	1199	0.08	Arginine	Twisting H-C2-H, H-C3-H, H-C4-H
84	1213	0.13	Arginine	Scissoring H-C1-C5 and twisting H-N1-H
85	1237	0.43	Aspirin	Vibration of hydrogen atoms in the plane of the ring
86	1258	0.01	Arginine	Wagging H-C2-H, H-C3-H
87	1270	0.40	Aspirin	Stretching C3-C2-O1
88	1290	0.05	Arginine	Wagging H-C2-H, H-C4-H and twisting H-C3-H
89	1304	0.20	Aspirin	Stretching O3=C9-O4, aromatic ring deformation, scissoring C9-O3-H
90	1319	0.13	Arginine	Twisting H-C3-H
91	1338	0.03	–	Twisting H-C2-H (arginine) and aromatic ring deformation (aspirin)
92	1339	0.28	–	Twisting H-C2-H (arginine) and aromatic ring deformation (aspirin)
93	1353	0.01	Arginine	Wagging H-C2-H, H-C4-H
94	1374	0.23	Arginine	Scissoring H-C3-H
95	1381	0.02	Arginine	Wagging H-C2-H, H-C3-H, H-C4-H
96	1399	0.06	Arginine	Scissoring H-N1-C1 and twisting H-N1-H
97	1403	0.02	Arginine	Scissoring H-N2-C5 and twisting H-N1-H
98	1409	0.20	Aspirin	Deformation of the aromatic ring in the ring plane
99	1429	0.24	Arginine	Scissoring H-N1-C1
100	1434	1.52	Aspirin	Vibration of hydrogen atoms in the plane of the ring
101	1453	0.22	Aspirin	Scissoring H-C3-H
102	1460	0.22	Aspirin	Scissoring H-C3-H
103	1468	0.10	Arginine	Scissoring H-C2-H
104	1479	0.09	Arginine	Scissoring H-C3-H, H-C4-H
105	1483	0.05	Aspirin	Vibration of hydrogen atoms in the plane of the ring
106	1496	0.03	Arginine	Scissoring H-C3-H, H-C4-H
107	1572	0.42	Aspirin	Vibration of hydrogen atoms in the plane of the ring and scissoring C9-O4-H
**108**	**1579**	**0.08**	**–**	**Scissoring H-N3-H and C5-N2-H, change in hydrogen bond length**
**109**	**1621**	**0.19**	**–**	**Scissoring H-N3-H, stretching N2-C5-N3, change in hydrogen bond length**
110	1647	0.61	–	Scissoring H-N1-H (arginine) and stretching O3=C9-C4 (aspirin)
111	1657	0.57	Arginine	Scissoring H-N3-H, H-N4-H
112	1669	0.35	Arginine	Scissoring H-N1-H
113	1677	0.23	Arginine	Scissoring H-N3-H, stretching N3-C5
114	1708	0.28	–	Scissoring H-N3-H, stretching N3-C5 (arginine) and stretching O2=C2 (aspirin)
115	1792	0.09	Arginine	Stretching C6-O1 and twisting H-N1-C1

Significant values are in [bold].

Calculated wavenumbers are presented with integer precision. The atomic number is indicated after the name of the element.

By superimposing theoretically calculated vibrational modes on the experimentally obtained spectra of platelets from a person who did not undergo therapy, and on the spectra of a person who underwent aspirin therapy, it can be determined that the changes occurring in the COX-1 receptor can be explained by the binding of aspirin to arginine at the position 376 in the receptor. It can be seen from the graphs that the change in the intensities and spectral shifts of the spectra during therapy correlates with the vibrational modes calculated theoretically.

## Discussions

The obtained spectra were analyzed based on the analysis of scientific literature. Most of the mentioned frequencies are experimentally obtained. From the obtained Figs. 6 and 7. The vibrational modes corresponding to CH_2_ vibrations, located at 1450 cm^−1^, 1451 cm^−1^, 1474 cm^−1^, 1475 cm^−1^ were revealed and reflected interaction of clopidogrel and arginine for H-O-C and CH_2_ bands. Vibration at 997 cm^−1^ correlates with stretching of 1-azacyclohexnene-3 and scissoring H–S-C (metabolite) and wagging NH (arginine) and can also can correlates with 1001 cm^−1^ characteristic of the aromatic ring experimental band of phenylalanine^12,19,20^. The spectral shift by 4 cm^-1^ in the case of clopidogrel therapy was revealed (the mode was detected at 997 cm^−1^). When considering the region 700–950 cm^−1^, several spectral differences of the groups under consideration were identified. Maximums 767, 905 describing vibrations of aromatic groups characteristic of the amino acids tyrosine, tryptophan and phenylalanine^12,19–21^, when exposed to different antiplatelet drugs, change in different ways. Spectral maximums at 1017 cm^−1^, 1019 cm^−1^ shows no correlation with the literature. Maximums 1450–1475 cm^−1^ and 1731–1764 cm^−1^ spectral regions correlate with vibrations in lipids^21^.

By superimposing theoretically calculated vibrational modes on the experimentally obtained spectra of platelets from a person who did not undergo therapy, and on the spectra of a person who underwent aspirin therapy, it can be determined that the changes occurring in the COX-1 receptor can be explained by the binding of aspirin to arginine at the position 376 in the receptor. It can be seen from the graphs that the change in intensities and spectral shifts of the spectra during therapy correlate with vibrational modes calculated theoretically. Table 3 shows the selected fluctuations for the binding sites of clopidogrel/aspirin to the platelet site, which can be potential biomarkers of the interaction of these compounds in the spectrum.

**Table 3:** Theoretical and experimental spectral characteristics of drug/metabolite receptor area. Calculated wavenumbers are presented with integer precision.

Vibrational mode number	Wave number, cm^−1^	Drug	Oscillation type
72	767	Clopidogrel	Rocking H-C2-H, H-C3-H, H-C2-H, H-C4-H (arginine) and scissoring C7-C8-C9, vibration of hydrogen atoms out of the plane of the aromatic ring (metabolite)
85	905	Clopidogrel	Twisting H-C2-H, H-C3-H, H-C4-H, stretching C5-N3, C5-N4 (arginine) and wagging H-O3-C9 (metabolite)
93	997	Clopidogrel	Stretching of 1-azacyclohexnene-3 and scissoring H–S-C6 (metabolite) and wagging N1-H(arginine)
96	1017	Clopidogrel	Stretching C2-C3-C4 (arginine) and stretching C1-O1-C2 (metabolite)
97	1019	Clopidogrel	Stretching C2-C3-C4 (arginine) and stretching C1-O1-C2 (metabolite)
146	1450	Clopidogrel	Scissoring H-O2-C6 (arginine) and scissoring H-C9-H (metabolite)
147	1451	Clopidogrel	Scissoring H-O2-C6 (arginine) and scissoring H-C9-H (metabolite)
151	1474	Clopidogrel	Scissoring H-C2-H, H-C3-H (arginine) and scissoring H-C1-H (metabolite)
152	1475	Clopidogrel	Vibration of hydrogen atoms in the plane of the aromatic ring (metabolite) and scissoring H-C2-H, H-C3-H (arginine)
153	1475	Clopidogrel	Scissoring H-C1-H (metabolite) and scissoring H-C2-H, H-C3-H (arginine)
163	1731	Clopidogrel	Stretching C2=O2 (metabolite) and scissoring H-N1-H (arginine)
164	1733	Clopidogrel	Stretching C2=O2, scissoring C9-O3-H (metabolite) and stretching C6=O1 (arginine)
165	1764	Clopidogrel	Stretching C2=O2 (metabolite) and stretching C6=O1, scissoring C9-O3-H (arginine)
32	450	Aspirin	Twisting H-N1-H, scissoring C1-C2-C3 (arginine) and scissoring C9-O4-H (aspirin)
33	465	Aspirin	Rocking H-N1-H (arginine) and rocking H-O4-C9 (aspirin)
35	492	Aspirin	Rocking H-N1-H (arginine) and rocking H-O4-C9 (aspirin)
37	510	Aspirin	Wagging H-N4-H
38	516	Aspirin	Wagging H-N4-H (arginine) and deformation of the aromatic ring in the ring plane (aspirin)
40	541	Aspirin	Deformation of the aromatic ring in the ring plane, scissoring O4-C9=O3 (aspirin) and rocking H-C3-H (arginine)
41	544	Aspirin	Scissoring O3=C9-C4 and aromatic ring stretching
45	608	Aspirin	Deformation of the aromatic ring in the ring plane, scissoring O4-C9 = O3
49	680	Aspirin	Aromatic ring stretching (aspirin) и rocking H-N3-H (arginine)
56	797	Aspirin	Wagging H-N3-H (arginine) and change in hydrogen bond length
58	814	Aspirin	Aromatic ring stretching (aspirin) and wagging H-N3-H (arginine)
72	1060	Aspirin	Rocking H-N3-H and change in hydrogen bond length
75	1087	Aspirin	Rocking H-N4-H, H-N3-H (arginine) and vibration of hydrogen atoms in the plane of the ring
91	1338	Aspirin	Twisting H-C2-H (arginine) and aromatic ring deformation (aspirin)
92	1339	Aspirin	Twisting H-C2-H (arginine) and aromatic ring deformation (aspirin)
108	1579	Aspirin	Scissoring H-N3-H and C5-N2-H, change in hydrogen bond length
109	1621	Aspirin	Scissoring H-N3-H, stretching N2-C5-N3, change in hydrogen bond length
114	1708	Aspirin	Scissoring H-N3-H, stretching N3-C5 (arginine) and stretching O2=C2 (aspirin)

During the experimental part, the spectra of human platelets were obtained without therapy, on aspirin therapy and on clopidogrel therapy. Platelet receptors and drugs that inhibit them have been investigated.

Using molecular docking, it was determined that the clopidogrel metabolite binds to arginine at position 137 in the P2Y12 receptor, and aspirin binds to arginine at position 376 in the COX-1 receptor. The correlation of theoretical and experimental data showed that the change in the SERS spectra of human platelets during therapy may be associated with a change in the conformation of amino acids (phenylalanine, tyrosine, and tryptophan). It was also determined that the changes that occur in the receptor during therapy can be explained by the binding of the drug to the receptors through arginine at different positions (137 for P2Y12 and 376 for COX-1). From the graphs obtained, it can be seen that the change in intensities and spectral shifts of the spectra during therapy correlate with vibrational modes calculated theoretically. Spectral shift at 1339 cm^−1^, 814 cm^−1^, 608 cm^−1^, 680 cm^−1^ correlates with aromatics was revealed. For 1339 cm^−1^ and 814 cm^−1^ the correlation with platelets aromatics in Trp were revealed^12^. 1708 cm^−1^ band lays near Amide I experimental band. Analyzing low-frequency 450–531 cm^−1^: 465 cm^−1^, 492 cm^−1^, 510 cm^−1^, 516 cm^−1^, 541 cm^−1^, 544 cm^−1^ bands corresponding for aspirin-arginine has been revealed and shows no direct correlation with experimental data, nevertheless 510 cm^−1^ and 516 cm^−1^ spectral bands correlates with –S–S, –C–S and –C–C vibrations^24^. Spectral bands at 544 cm^−1^, 797 cm^−1^, 1060 cm^−1^, 1087 cm^−1^, 1579 cm^−1^. 1621 cm^−1^ characterize hydrogen bonding in the complex and also can be potential biomarkers of therapy response for aspirin. Aspirin can form the assigned H-bonding interaction with different groups, including COX-1 receptor^22,23^. This fact, proved theoretically and experimentally in our paper, confirms the correctness of receptor-drug interaction DFT simulation (Supplementary Information).

## Conclusions

The authors perform molecular docking and mathematical DFT simulation for antiplatelet drug and the target platelet receptor/ferment interaction in the limited area. The Raman bands, corresponding to the drug, aspirin/clopidogrel and its interaction in the binding site have been revealed. Vibrational modes shown in Table 3 can be potential biomarkers of clopidogrel/aspirin interaction with the corresponding platelet receptors (P2Y12/COX-1). As a result, theoretical Raman spectra of the drug-receptor interaction area were obtained. The theoretical data were compared with the experimental SERS results. Characteristics bands corresponding to metabolite/ferment and antiplatelet drug vibrations were clarified. The prospects of obtaining results for pathologies based on platelet conformations during cardiovascular diseases have been demonstrated.

### Supplementary Information

Supplementary Information.

## References

[1] Roth GA, Mensah GA, Johnson CO, Addolorato G, Ammirati E, Baddour LM (2020). Global burden of cardiovascular diseases and risk factors, 1990–2019: Update from the GBD 2019 study. J. Am. Coll. Cardiol..

[2] Ouriel K, Donayre C, Shortell CK, Cimino C, Donnelly J, Oxley D (1991). The hemodynamics of thrombus formation in arteries. J. Vasc. Surg..

[3] Hartmann J, Hussein A, Trowitzsch E, Becker J, Hennecke KH (2001). Treatment of neonatal thrombus formation with recombinant tissue plasminogen activator: Six years experience and review of the literature. Arch. Dis. Child. Fetal Neonatal Ed..

[4] Wang GW, Yao HL, He BJ, Peng LX, Li YQ (2007). Raman micro-spectroscopy of single blood platelets. Guang pu xue yu Guang pu fen xi Guang pu.

[5] Zyubin A, Rafalskiy V, Tcibulnikova A, Matveeva K, Moiseeva E, Tsapkova A (2020). Dataset of human platelets in healthy and individuals with cardiovascular pathology obtained by Surface-enhanced Raman spectroscopy. Data Brief.

[6] Tah B (2014). Quantum-mechanical DFT calculation supported Raman spectroscopic study of some amino acids in bovine insulin. Spectrochim. Acta Part A: Mol. Biomol. Spectrosc..

[7] Kausar N (2009). Vibrational spectroscopy and DFT calculations of the di-amino acid peptide L-aspartyl-L-glutamic acid in the zwitterionic state. Phys. Chem. Chem. Phys..

[8] Ma H (2019). Surface-enhanced Raman scattering for direct protein function investigation: Controlled immobilization and orientation. Anal. Chem..

[9] Zdaniauskienė A (2022). Shell-isolated nanoparticle-enhanced Raman spectroscopy for probing riboflavin on graphene. Materials.

[10] Mahar N (2021). Fast and sensitive detection of Procainamide: Combined SERS and DFT modeling studies. J. Mol. Liquids.

[11] Zyubin A (2022). Spectral homogeneity of human platelets investigated by SERS. Plos One.

[12] Zyubin A (2020). Surface-enhanced Raman spectroscopy for antiplatelet therapy effectiveness assessment. Laser Phys. Lett..

[13] Liu C (2019). Pharmacokinetics and pharmacokinetic/pharmacodynamic relationship of vicagrel, a novel thienopyridine P2Y12 inhibitor, compared with clopidogrel in healthy Chinese subjects following single oral dosing. Eur. J. Pharmaceut. Sci..

[14] Anaconda website (2023, accessed 15 Mar 2023). https://anaconda.org/salilab/modeller. Schrodinger: Website (2023, accessed 15 Mar 2023). https://pymol.org/2/.

[15] Frisch MJ, Trucks GW, Schlegel HB (2016). Gaussian16, Revision ().

[16] Becke AD (1993). Development of the Colle-Salvetti correlation-energy formula into a functional of the electron density. J. Chem. Phys..

[17] Yoshida H (2002). A new approach to vibrational analysis of large molecules by density functional theory: Wavenumber-linear scaling method. J. Phys. Chem. A.

[18] Fayfel A. B., Berezin K. V. & Nechaev V. V. A program for modeling and visualizing vibrational IR and Raman spectra based on quantum mechanical calculation data. In *Problems of Optical Physics. Problems of Optical Physics*. (Saratov: Publishing house of the State Scientific Center “College, 2003).

[19] HORIBA: Website (2023, accessed 16 Mar 2023). https://www.horiba.com.

[20] Zhu G, Zhu X, Fan Q, Wan X (2011). Raman spectra of amino acids and their aqueous solutions. Spectrochim. Acta A Mol. Biomol. Spectrosc..

[21] García-Rubio DL (2019). Analysis of platelets in hypertensive and normotensive individuals using Raman and Fourier transform infrared-attenuated total reflectance spectroscopies. J. Raman Spectrosc..

[22] Zeinalipour-Yazdi CD (2022). A DFT study of the interaction of aspirin, paracetamol and caffeine with one water molecule. J. Mol. Model..

[23] Lei J (2015). Mechanistic insights into a classic wonder drug aspirin. J. Am. Chem. Soc..

[24] El-Hag DA, Dahab AA (2016). Identification and characterisation of disulphide bonds in therapeutic proteins by using Raman Spectroscopy. Adv. J. Pharm. Life Sci. Res..

